# A Unique Enoxaparin Derived from Bovine Intestinal Heparin: A Single Purification Step of the Starting Material Assures a Bovine Enoxaparin Like the Standard from Porcine Origin

**DOI:** 10.1021/acsomega.4c02128

**Published:** 2024-05-10

**Authors:** Stephan N. M. C. G. Oliveira, Francisco F. Bezerra, Adriana A. Piquet, Rodrigo A. Sales, Gabrielly C. T. Valle, Nina V. Capillé, Ana M. F. Tovar, Paulo A. S. Mourão

**Affiliations:** Laboratório de Tecido Conjuntivo, Hospital Universitário Clementino Fraga Filho and Instituto de Bioquímica Médica Leopoldo de Meis, Universidade Federal do Rio de Janeiro, Rio de Janeiro 21941-913, Brazil

## Abstract

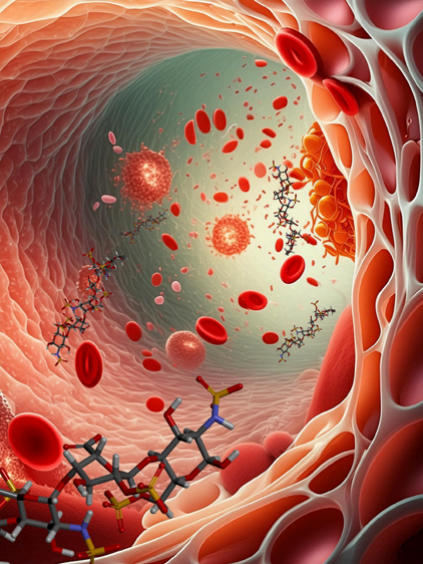

Low-molecular-weight heparin represent a significant advancement in anticoagulant therapy with enoxaparin being a prominent example obtained exclusively through the fragmentation of porcine intestinal heparin. However, escalating demand and limited resources have raised concerns about enoxaparin supplementation. The current challenge involves exploring alternative heparin sources for large-scale enoxaparin production with bovine intestinal heparin emerging as a promising option. Our study demonstrates that enoxaparin derived from the available bovine heparin preparation differs significantly from the reference compound. Yet, the implementation of a straightforward purification step yields a preparation termed “high-anticoagulant bovine heparin”. Fragmentation of this purified product through β-elimination produces enoxaparin akin to the standard from a porcine origin. To ensure physicochemical similarity, we employed various spectroscopic, enzymatic, and chromatographic tests to compare the new bovine-derived enoxaparin with the original porcine compound. Biological activity was confirmed through *in vitro* coagulation assays and assessments using an animal model of venous thrombosis. Our study affirms that the β-elimination reaction cleaves the bovine heparin chain without preferential breaks in regions with different sulfation patterns. Additionally, we scrutinized decasaccharides purified from enoxaparin preparations, providing a comprehensive demonstration of the similarity between products obtained from porcine and bovine heparin. In summary, our findings indicate that an enoxaparin equivalent to the original porcine-derived product can be derived from bovine heparin, given that the starting material undergoes a simple purification step.

## Introduction

The introduction of low-molecular-weight heparins (LMWHs) marked a significant moment in anticoagulant therapy, leading to substantial advantages, including predictable responses, subcutaneous administration, and minimal side effects.^1,2^ These compounds have proven to be valuable alternatives to unfractionated heparins (UFHs). Despite these advantages, the broader adoption of LMWHs are hindered by their significant cost, limiting accessibility, particularly in countries such as Brazil.^3^ Here, subcutaneous UFH administration persists, driven by economic constraints and raising questions about its effectiveness. Enoxaparin, among various LMWHs, stands out as the most widely used worldwide.^4^

LMWHs are traditionally derived from porcine intestinal heparin (HPI) through a meticulously controlled chemical such as a ß-elimination reaction. This process involves two key steps: (i) cleavage of the polysaccharide backbone and (ii) hydrolysis of residual esters under alkaline conditions, contributing to the high production costs and limited availability.^5,6^ However, there is significant debate regarding the equivalence of UFHs from alternative sources to HPI, especially in their application to LMWH production, such as enoxaparin.^7−9^ Regulatory agencies such as the FDA have been encouraging the search for new sources of heparin.^10^ Heparin from bovine intestinal heparin (HBI) emerges as a promising source due to its potential for large-scale preparation, albeit notable differences in disaccharide composition and anticoagulant effects compared to HPI.^11,12^

However, the transition from HPI to HBI presents great challenges. Notable differences in disaccharide composition and the anticoagulant effect between HPI and HBI have been observed. The most striking distinction lies in the higher content of α-GlcNH units *N*-sulfated but 6-desulfated in HBI than in HPI.^13−15^ Nevertheless, some studies have reported the production of enoxaparin from HBI, although without a comprehensive structural analysis coupled with effectiveness tests in biological assays.^11,12^ The absence of information regarding structural differences prompted our investigation into the production of enoxaparin from HBI and its biological activity.

**Figure 1:**
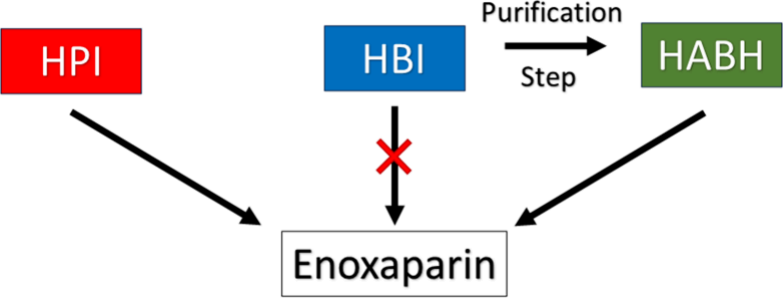
Outline of the experimental approach employed in this study for the preparation of bovine enoxaparin. Initially, we generated enoxaparin through a β-elimination reaction of pharmaceutical preparations of porcine heparin (HPI) and bovine heparin (HBI). The products obtained from these two heparins exhibited marked differences. As an alternative, we prepared high-anticoagulant bovine heparin (HABH) from HBI using a single step of anion exchange chromatography. The enoxaparin generated from HABH showed similarity to the standard product from porcine origin in physicochemical and biological characteristics. Only minor differences in disaccharide composition were observed, but they had no impact on the biological activity. These differences may serve as a fingerprint to distinguish bovine enoxaparins from porcine enoxaparins.

Our study addresses this issue by examining enoxaparin derived from HBI. Initial results revealed significant structural and biological differences compared to the standard HPI product, as expected. Introducing a single purification step creates a high-anticoagulant bovine heparin (HABH), serving as a suitable source to produce enoxaparin equivalent to the gold standard from porcine origin. Figure 1 summarizes the approach used in this work. Although there are slight disparities in the disaccharide composition between enoxaparins from HABH and HPI, these variations do not significantly impact biological activity. These differences can potentially serve as markers to distinguish between sources of enoxaparin (porcine vs bovine).

Our study also unveils insights into the β-elimination reaction, a crucial step in generating enoxaparin from UFH. Contrary to expectations, the reaction does not exhibit preferential action on α-GlcNH units with specific sulfation patterns. It cleaves the heparin chain, affecting α-GlcNH units equally, whether they have 6-sulfation. Further analysis of decasaccharides from these preparations reaffirms the similarity between products obtained from HPI and HABH.

In summary, our research represents a pioneering advancement in identifying alternative sources for enoxaparin production. By integrating rigorous structural analysis with assessments of the biological impact, we contribute to the development of safe, effective, and economically viable anticoagulant products, paving the way for broader accessibility to LMWHs.

## Results

### Physicochemical and Biological Characterization of Enoxaparins Obtained from Bovine Heparins

#### Identification of Constituent Monosaccharides by NMR Spectroscopy

**Figure 2:**
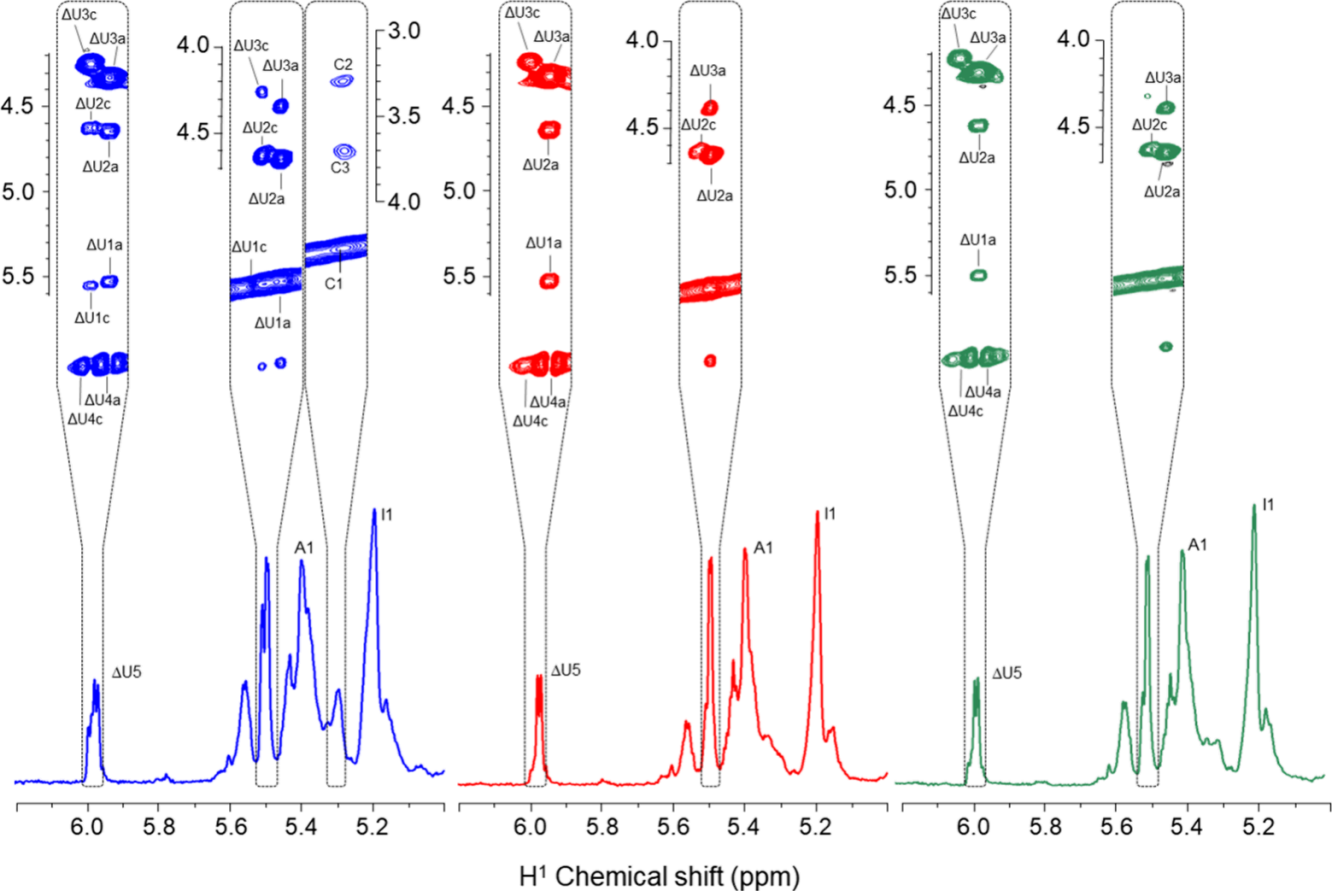
1D ^1^H NMR spectra and strips of 2D ^1^H–^1^H phase-sensitive TOCSY spectra (on the top of the panels) of enoxaparins obtained from HBI (A, blue), HPI (B, red), and HABH (C, in green). ΔUa and ΔUc are signals of Δ4,5UA units linked to α-GlcN,6-diS and α-GlcNS, respectively. A1 and C1 are anomeric signals of the α-GlcN,6-diS and α-GlcNS, respectively. I1 is the anomeric signal of α-IdoA.

The pharmaceutical preparations of HBI and HPI were subjected the β-elimination procedure, as recommended for the generation of enoxaparin.^6^ The resulting products were analyzed using 1D and 2D NMR techniques. While the 1D ^1^H NMR spectra revealed similarities between the two enoxaparins (Figure 1SA,B), significant differences were observed in certain regions, as indicated by the dotted rectangles in Figure 2A,B.

More comprehensive analysis using 2D ^1^H–^1^H TOCSY spectra (Figure 2S) revealed these distinctions between enoxaparins obtained from HBI and HPI, as illustrated in the inset within the upper part of Figure 2A,B. Enoxaparin obtained from HBI, as expected, contains an α-GlcNS spin system (named as C) more intense than originating from HPI. The chemical shift values of this system are equivalent to those previously reported for HBI.^15^ Another observation is the presence of two spin systems of unsaturated hexuronic acid (Δ4,5UA) at the nonreducing ends of the molecule, designated as Ua and Uc, attributed to units linked to α-GlcN,6-diS and α-GlcNS, respectively.

These findings align with observations that pharmaceutical preparations of HBI have high proportions of 6-desulfated α-GlcNS, similarly reflected in bovine enoxaparin.^15,16^ To address this limitation, an HBI obtained after an additional purification step (HABH) was utilized. Enoxaparin prepared from HABH exhibited a 1D ^1^H (Figure 2 and Figure 1SB,C) and 2D ^1^H–^1^H TOCSY (strips in Figure 2B,C,Figure 2S) NMR spectra like the product obtained from HPI.

**Figure 3:**
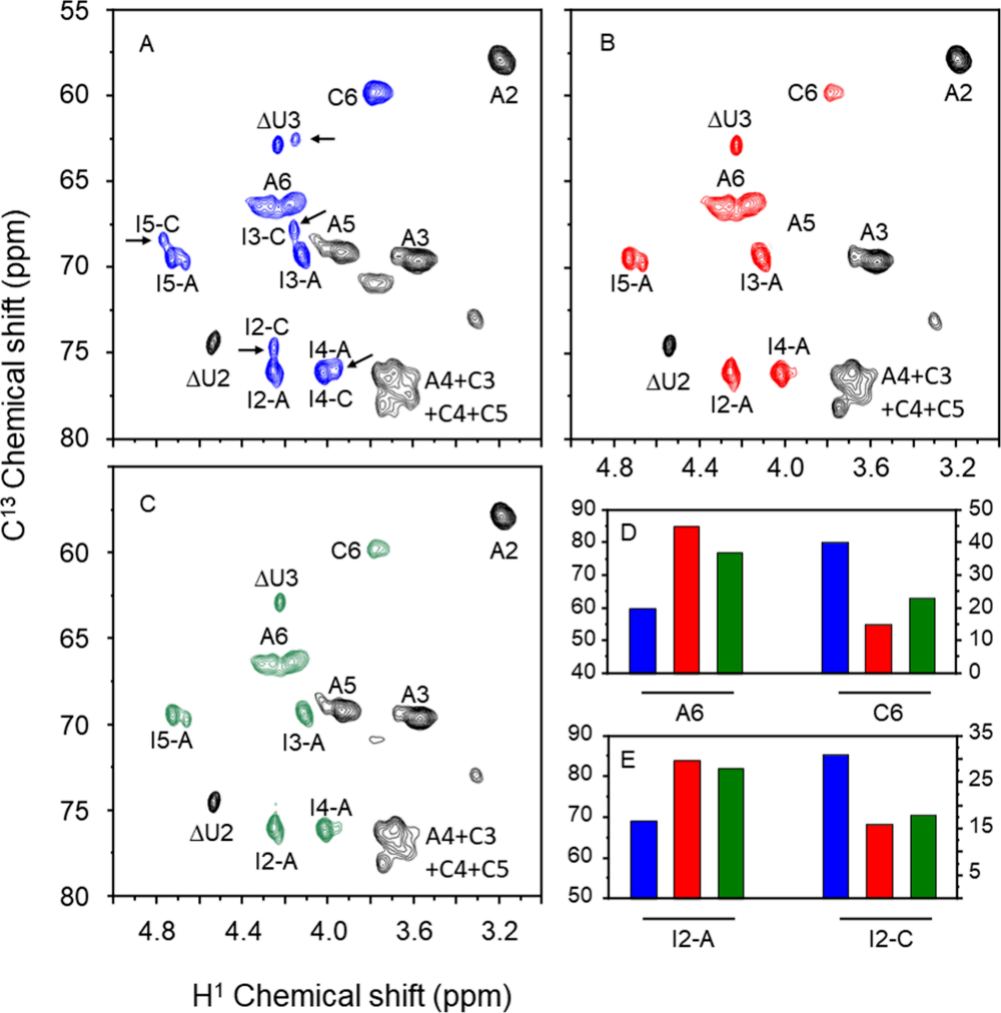
^1^H–^13^C HSQC spectra in the region between 3–5/55–80 ^1^H/^13^C ppm of the enoxaparins obtained from HBI (A), HPI (B), and HABH (C). Panels D and E show the integrals (as % of total) of A6/C6 and I2-A/I2-C signals in the spectra of the three enoxaparins. Signals showing major differences among the three types of enoxaparins are colored and indicated by arrows. C6 and A6 are C6/H6 signals of α-GlcN,6-diS and α-GlcNS units, respectively. I2, I3, I4, and I5 are signals of α-IdoA2S, linked either to α-GlcN,6-diS, denoted as set A, or to α-GlcNS, denoted as set C.

The analysis through 2D ^1^H–^13^C HSQC spectra revealed differences between enoxaparins obtained from the three types of heparin preparations in the anomeric region (Figure 3S). The quantification is presented in Table 1. There were notably high levels of α-GlcN without 6-sulfation in enoxaparin from HBI (HBI > HABH > HPI). Additionally, other minor differences were observed, particularly in the 2-sulfation content of α-IdoA (HABH > HBI/HPI), while the unsaturated hexuronic acid units (ΔU1) at the nonreducing terminals appeared in similar proportions across the three types of enoxaparins, suggesting comparable degrees of cleavage.

**Table 1:** Monosaccharide Composition of Enoxaparins Obtained from Different Types of Heparins Based on ^13^C–^1^H HSQC Spectra

signal	structure	HBI	HPI	HABH
(A) α-Glucosamine units				
A	α-Glc*N*,6-diS-[α-IdA2S]	39.5 ± 0.40	49.2 ± 0.78	51.5 ± 0.41
A′	α-Glc*N*Ac-[β-GlcA]	2.40 ± 0.42	2.90 ± 0.42	1.34 ± 0.49
B	α-GlcNS-[α-IdA]	12.5 ± 1.14	11.3 ± 1.13	2.15 ± 0.97
C	α-Glc*N*S-[α-IdA2S]	15.0 ± 0.28	12.4 ± 1.12	12.4 ± 0.49
D	α-Glc*N*S-[β-GlcA]	20.9 ± 1.70	14.4 ± 1.32	19.7 ± 1.41
A-r	A-red	9.65 ± 0.28	9.70 ± 0.28	12.8 ± 0.29
(B) α-Iduronic acid				
I	α-IdA2S	47.0 ± 5.40	45.1 ± 1.92	52.8 ± 5.04
J	α-IdA-[α-Glc*N*,6S]	0.84 ± 0.10	4.50 ± 1.83	1.84 ± 0.63
J′	α-IdA-[α-GlcN]	1.18 ± 0.06	0.72 ± 0.24	0.80 ± 0.36
	∑ α-IdA	49.0 ± 0.57	50.3 ± 0.16	55.4 ± 0.60
E	AU1	28.3 ± 0.36	29.1 ± 0.20	27.3 ± 1.38
K′	β-GlcA-[α-Glc*N*,3,6-triS]	1.15 ± 0.35	3.05 ± 0.49	2.69 ± 0.85
K	β-GlcA-[α-Glc*N*Ac]	7.68 ± 0.39	6.06 ± 0.19	5.92 ± 2.86
L	β-GlcA2S-[?]	5.20 ± 0.99	1.25 ± 0.35	1.57 ± 1.09
M	β-GlcA-[α-Glc*N*S]	8.80 ± 0.85	10.7 ± 0.84	8.58 ± 0.41
	∑ β-GlcA	17.6 ± 0.89	19.8 ± 1.15	17.2 ± 4.12
	N-sulfation^a^	75.4 ± 1.08	76.0 ± 0.72	83.7 ± 0.23
	6-sulfation^b^	55.2 ± 2.79	85.7 ± 0.63	76.0 ± 3.61
	N-acetylation^a^	2.40 ± 0.42	2.90 ± 0.42	1.34 ± 0.49
	2-sulfation^a^	47.0 ± 5.4	45.1 ± 1.92	52.7 ± 5.04

a Calculated using integrals of the anomeric signal.

b Calculated based on the H6/C6 integrals of 6-sulfated and nonsulfated α-GlcN. Refer to signals A6 and C6.

The ^1^H–^13^C HSQC spectra highlighted interesting observations in the region between 3–5/55–80 (^1^H/^13^C) ppm, indicating the similarity of enoxaparins obtained from HPI and HABH and their distinctions from that obtained from HBI (see colored signals in Figure 3A–C). The first difference observed is in the intensity of the A6 signal compared to C6 (HPI > HABH > HBI, integrals in Figure 3D). A6 and C6 correspond to the C6/H6 of 6-sulfated and nonsulfated α-GlcNH units, respectively. Another set of signals is related to the α-Ido2S units, as indicated by shifts observed in the signals neighboring α-GlcNS compared to those linked to α-GlcN,6-diS (3E). These shifts serve to differentiate the enoxaparins obtained from HPI/HABH from that of HBI.

In summary, the comprehensive analysis based on NMR spectroscopy underscores the similarity of enoxaparins obtained from HABH and HPI, while enoxaparin derived from HBI exhibits significant differences, primarily due to the high content of α-GlcNS units without sulfation at position 6. These findings pave the way for further exploration and refinement to achieve the desired similarity between enoxaparins derived from different heparin sources.

#### Analysis of the Disaccharides Formed by Digestion of the Enoxaparins with Heparitinases

**Figure 4:**
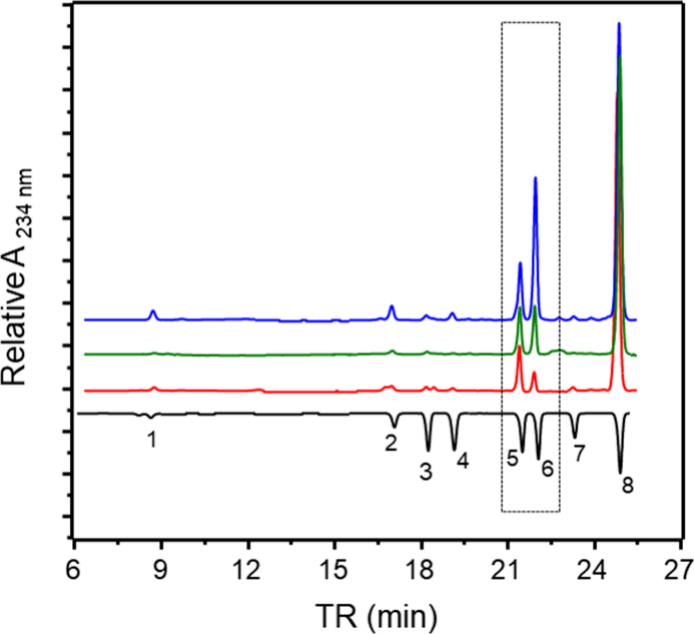
Chromatograms of the disaccharides formed by digestion of the enoxaparins generated from HBI (in blue), HPI (in red), and HABH (in green) with a mixture of heparinases I, II, and III. The disaccharides showing more prominent differences between bovine and porcine enoxaparins are indicated by the dotted rectangle. Elution of standard disaccharides is shown as negative signals in the lower panel and enumerated as in Table 2.

We employed heparitinases digestion to analyze the constituent units of the three enoxaparins. The disaccharides resulting from the enzyme digestion were separated using ion exchange chromatography coupled with an HPLC system (Figure 4). The quantitative values are presented in Table 2. As expected, the predominant product from the three enoxaparins is the trisulfated disaccharide (ΔUA2S-GlcNS,6S). No difference was observed between the disaccharide composition of the enoxaparin produced in our laboratory (HPI-C, Table 2) and the standard from USP (HPI-A) or a generic product available in Brazil (HPI-B). In contrast, the composition of the enoxaparin derived from HBI, HPI, and HABH differs significantly. The primary distinction is the proportion of the disulfated disaccharide ΔUA2S-GlcNS (HBI > HABH > HPI), potentially serving as a fingerprint that distinguishes enoxaparins derived from the two sources (HABH and HPI). Other subtle differences may be observed, but less prominent.

**Table 2:** Disaccharide Compositions of Enoxaparins Obtained from Different Types of Heparins

	enoxaparins				
disaccharides	HBI	HPI-A^a^	HPI-B^b^	HPI-C^c^	HABH
1 ΔUA-GlcNAc	1.9	3.16	3.66	2.79	2.82
2 ΔUA-GlcNS	3.06	2.23	2.52	2.22	2.73
3 ΔUA-GlcNAc,6S	1.24	3.70	3.70	2.32	0.89
4 ΔUA2S-GlcNAc	1.28	1.43	1.53	1.70	1.28
5 ΔUA-GlcNS,6S	**10.37**	**13.16**	**12.96**	**11.04**	**10.68**
6 ΔUA2S-GlcNS	**22.96**	**6.58**	**6.73**	**8.66**	**14.58**
7 ΔUA2S-GlcNAc,6S	0.79	1.56	1.69	1.36	0.82
8 ΔUA2S-GlcNS,6S	**58.39**	**68.19**	**67.21**	**69.91**	**66.21**
					
2S	83.42	77.76	77.16	81.63	82.88
6S	70.79	86.61	85.56	84.63	78.60
NS	94.78	90.16	89.42	91.83	94.19
NAc	5.21	9.84	10.58	8.17	5.81

a Biossimilar enoxaparin.

b Enoxaparin USP standard.

c Enoxaparin prepared in our laboratory.

#### Molecular Mass Distribution

**Figure 5:**
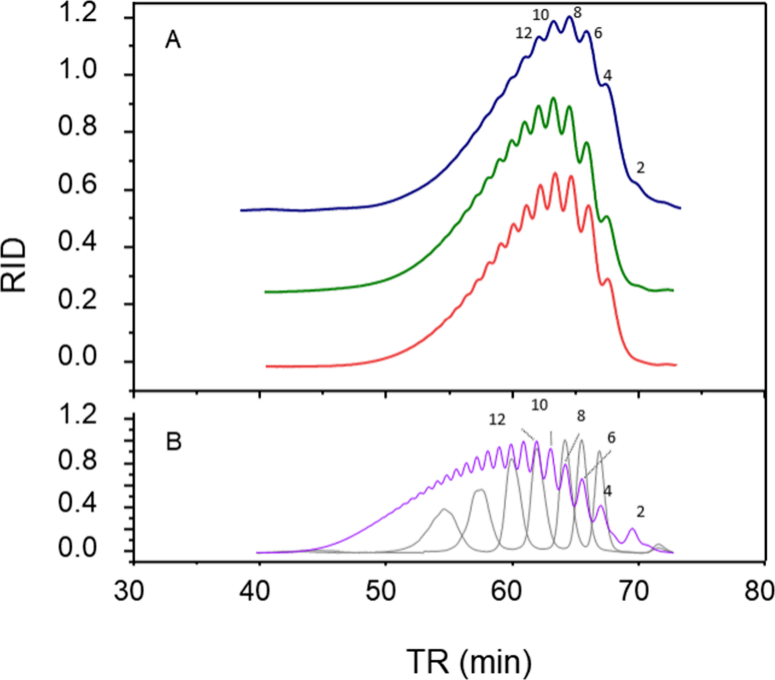
Profiles of size exclusion chromatography on TSK 3000 and TSK 2000 columns of enoxaparins derived from HBI (blue), HPI (red), and HABH (green) (panel A). Panel B shows elution of the molecular weight standard from NIBSC (purple) and USP (broken lines). The numbers in the panel indicate the degree of polymerization of the oligosaccharides.

Enoxaparin preparations contain mixtures of oligosaccharides that are separated by gel filtration chromatography (Figure 5). The chromatogram of enoxaparin derived from HBI is like others, containing the same peaks but differing in their quantification. The peaks of oligosaccharides formed from HBI are also less resolved compared to the product obtained from HPI and HABH (chromatograms in blue vs green/red in Figure 5A), suggesting that they may have a more heterogeneous composition. Furthermore, the molecular mass distribution, obtained by area quantification of the chromatogram peaks, of the enoxaparins obtained from HBI does not meet the requirements for this type of LMWH, due to a high content of components with molecular mass below 2000 Da (Table 3). These results confirm the dissimilarity between products generated from HBI and HPI by the β-elimination. When HABH was used instead of HBI, we noted an increased resolution of the oligosaccharides in gel chromatography. Furthermore, all the pharmaceutical requirements for enoxaparin were achieved (green vs red, Figure 5A and Table 3).

**Table 3:** Molecular Mass Distribution of Enoxaparin Preparations Obtained from Different Types of Heparins^a^

	enoxaparins					
parameter	HABH-A	HABH-B	HBI-A	HBI-B	HPI-A	HPI-B
Mw	4580.1	4486.9	4004.1	3996.0	4284.9	4405.5
Mn	3281.9	3325.3	2669.4	2626.0	3107.0	3095.0
PD	1.4	1.4	1.5	1.5	1.4	1.4
M <2000 Da	13.2	13.0	22.4	23.4	14.7	16.3
M 2000–8000 Da	75.7	75.0	69.9	68.1	76.6	74.0
M >8000 Da	11.1	12.0	7.8	8.5	8.7	9.7

a A and B are batches of enoxaparin prepared individually.

#### Anticoagulant and Antithrombotic Activities

**Figure 6:**
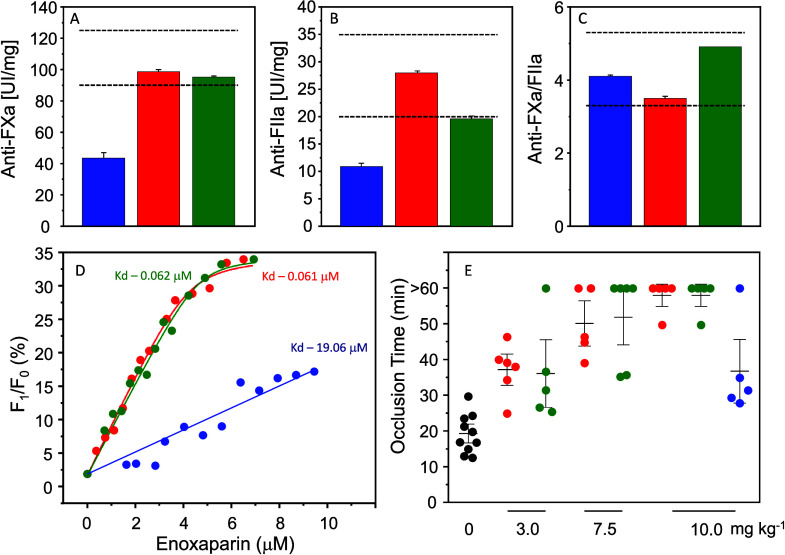
Biological activities of the enoxaparins derived from HBI (blue), HPI (red), and HABH (green). Panels A and B show anti-FXa and anti-FIIa activities of the enoxaparins, while the ratios between these two activities are displayed in panel C. Broken lines indicate the limits of these activities recommended by USP. Panel D shows the titration of an antithrombin solution by increasing concentrations of the enoxaparins, and the *K*_d_ values are indicated in the panel. Panel E shows the antithrombotic effect of the enoxaparins based on the evolution of thrombus induced in the mesentery veins by laying a filter paper soaked with 8% ferric chloride and observed by an intravital microscope for 60 min. The results are expressed as the time required for the total obstruction of the vein.

In the standardized coagulation assays, enoxaparin obtained from HBI exhibits approximately half of the anticoagulant activity compared to the product obtained from HPI, as evidenced in both anti-FXa (43.3 ± 3.2 vs 98.7 ± 1.0 IU mg^–1^) and anti-FIIa (10.8 ± 2.3 vs 28.0 ± 0.7 IU mg^–1^) assays (Figure 6A–C). In contrast, the product from HABH demonstrates activity compatible with or similar to those found in enoxaparin from HPI (95.2 ± 0.4 and 19.6 ± 1.0 IU mg^–1^ of anti-FXa and FIIa activity, respectively). The upper and lower thresholds of the pharmacopeia are marked by dashed lines in Figure 6A–C. The HBI-derived enoxaparin falls below the established threshold.

Another approach employed to evaluate the biological effect of enoxaparins was to establish their affinity for antithrombin, the primary target molecule of heparin. This was achieved through titration based on the change in intrinsic serpin fluorescence (Figure 6D). Once again, the study revealed the similarity between enoxaparins obtained from HABH and HPI, while the product derived from HBI exhibited reduced affinity for serpin (*K*_d_ values HABH = HPI < HBI).

Finally, the three types of enoxaparins underwent testing in an experimental model of venous thrombosis in mice (Figure 6E). The assessment of thrombus formation via intravital microscopy demonstrated a similar effect of enoxaparins obtained from HABH and HPI, whereas the product formed from HBI exhibited significantly less activity.

### Analysis of the Oligosaccharides Generated from Bovine and Porcine Heparin by the β-Elimination Reaction

#### β-Elimination Reaction Does Not Cleave Preferentially α-GlcNH Units with Distinct Sulfation Patterns

**Figure 7:**
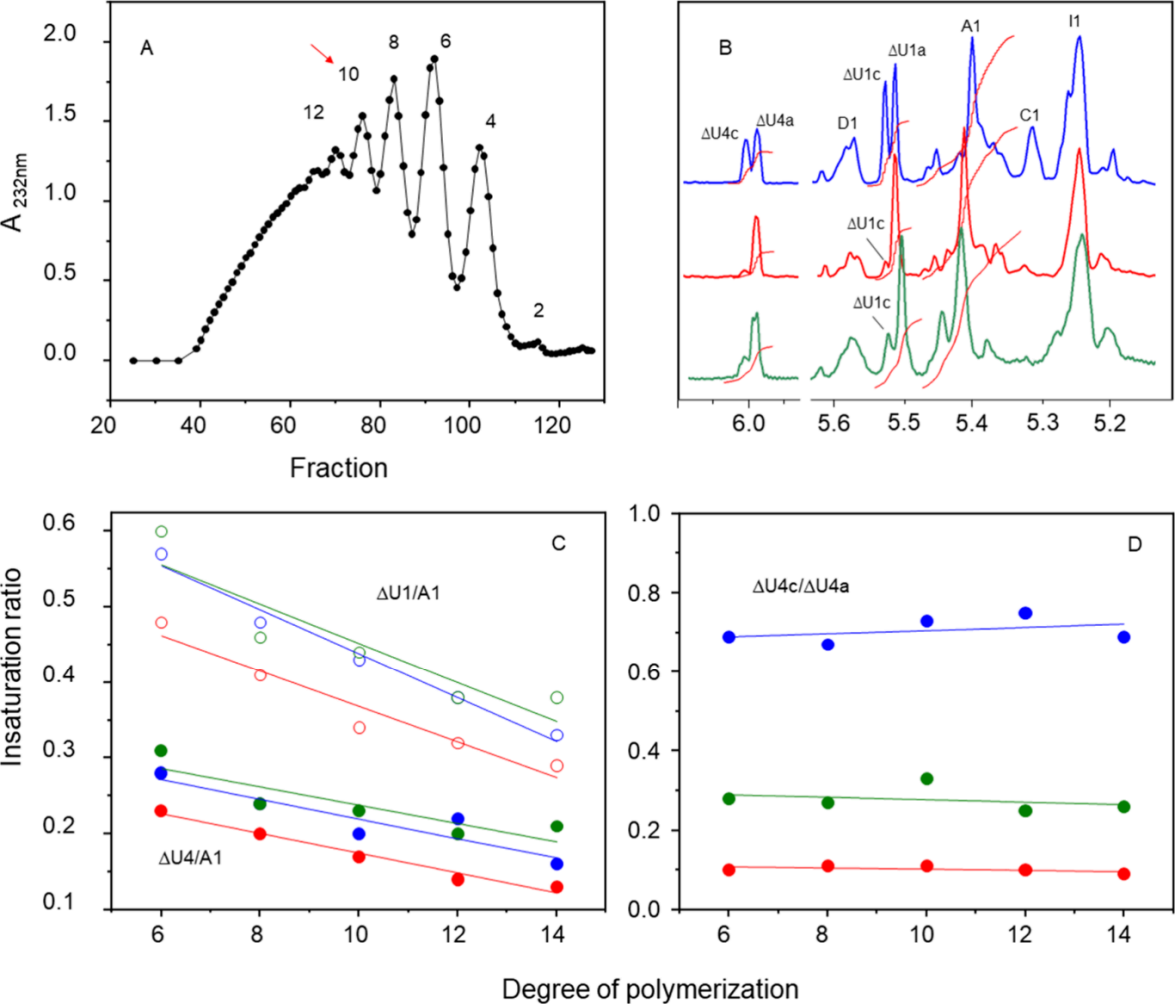
Fractionation and analysis of the constitutive oligosaccharides from enoxaparin obtained from HBI (blue), HPI (red), and HABH (green). Panel A shows a typical preparative gel-permeation chromatography of enoxaparin from HPI on a Bio-Gel P-10 column followed by A_232_ nm. The numbers in the panels indicate the DP of the oligosaccharides. Panel B shows the 1D ^1^H NMR spectra of the decasaccharides (indicated by the red arrow in panel A) obtained from the three types of enoxaparins. See Figure 2 for the nomenclatures of the signals. Panels C and D show the ratios of signal integrals, as indicated in the panels.

The oligosaccharides obtained from HBI, HPI, and HABH were separated through preparative gel filtration chromatography (Figure 7A) and analyzed by ^1^H NMR spectroscopy. The spectra of the anomeric region obtained from decasaccharides are shown in Figure 7B, while full spectra of then and those of the other oligosaccharides are presented in Figure S4. Three distinct regions of the spectra are easily identified and can be utilized to quantify components of the oligosaccharide structure. Two of these regions correspond to the signals ΔU4c/ΔU4a and ΔU1c/ΔU1a, representing H4 and H1 of the Δ4,5UA units linked to α-GlcNS (ΔU4c and ΔU1c) and α-GlcN,6-diS (ΔU4a and ΔU1a), respectively. The third crucial region of the spectra is denoted as A1, housing the anomeric protons of the α-GlcNS,6-diS units. Integration of these regions in the 1D ^1^H NMR spectra of the oligosaccharides formed from HBI, HPI, and HABH is depicted in Figure 7C,D. The integrals of H1 and H4 of the Δ4,5UA units decrease with the increase in the degree of polymerization (DP), as expected (Figure 7C). More significantly, the relative intensities of the ΔU4c and ΔU4a signals do not vary as a function of the DP (Figure 7D).

#### Analysis of the Decasaccharides Purified from the Three Types of Enoxaparins

**Figure 8:**
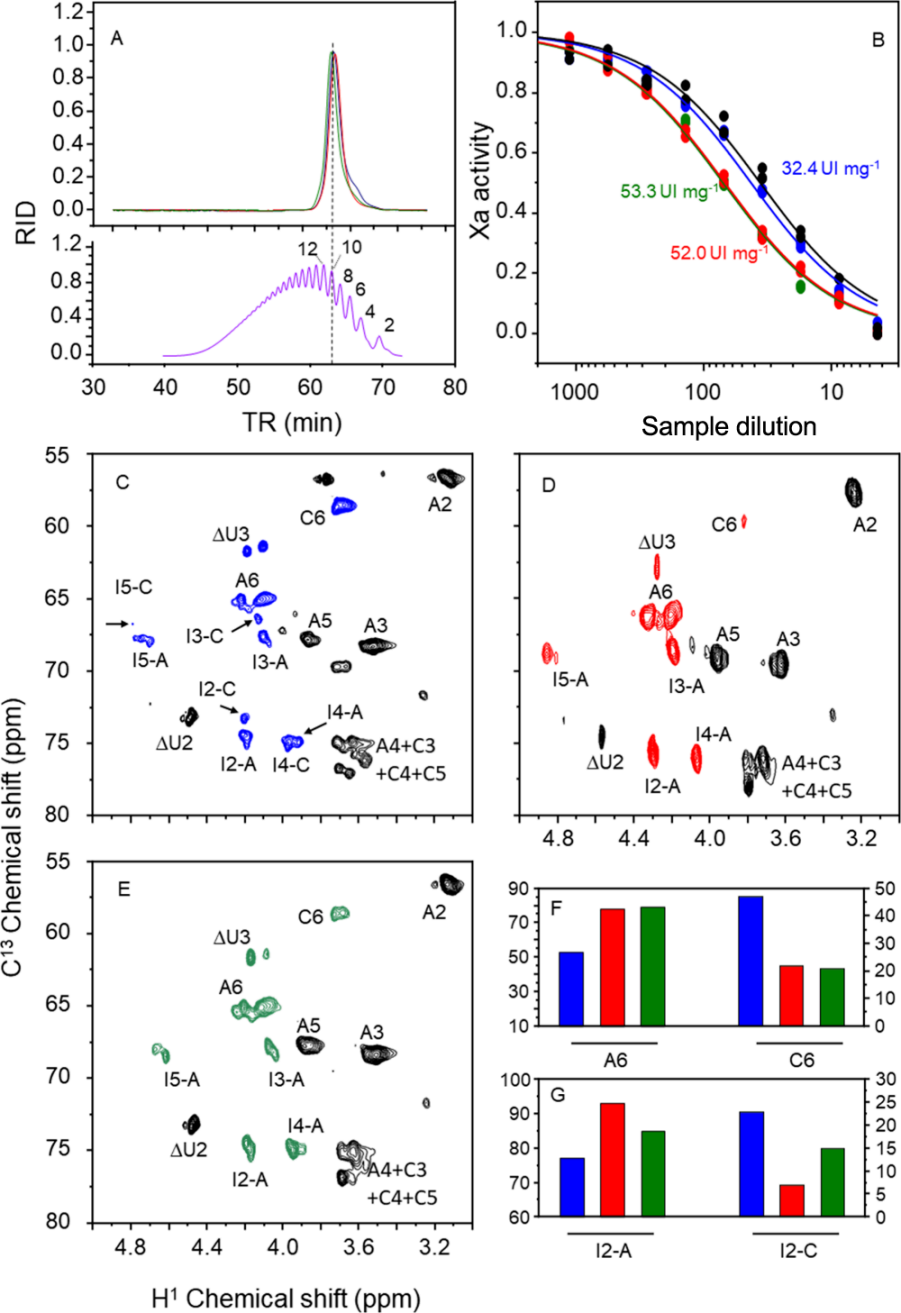
Analysis of the decasaccharides obtained from enoxaparins derived from HBI (blue), HPI (red), and HABH (green). Panel A shows the analysis of the decasaccharides on an analytical gel-permeation column (as shown in Figure 4). The lower panel indicates the elution of the molecular size standard from NIBSC, and the numbers in the panel indicate the DP. Panel B shows the anti-FXa activity of the decasaccharides purified from the three types of enoxaparins, and the numbers indicate the activity as IU mg-1. Results in black circles are from the LMWH standard from NIBSC. Panels C and D show the ^1^H–^13^C HSQC spectra in the region between 3–5/55–80 ^1^H/^13^C ppm of the decasaccharides derived from HBI (panel C), HPI (panel D), and HABH (panel E). The arrows in panel C indicate the signals of α-Ido2S units linked to α-GlcNS. Panels F and G indicate the integration of signals A6, C6, I2-A, and I2-C in the spectra of the three decasaccharides.

Initially, the three decasaccharides underwent analytical gel filtration chromatography to ensure the purity of the preparations, displaying a single peak (Figure 8A). In Figure 8B, it is evident that the decasaccharide derived from HBI exhibits significantly reduced anticoagulant action compared to those obtained from other heparins (HBI < HABH = HPI). Once again, we observed the characteristic intense signal of C6/H6 from the 6-desulfated GlcNS (C6) in the HBI-derived decasaccharide, while this signal is more discrete in the products from HPI and HABH (colored signals in the panels). Similarly, the signals from the 2-sulfated iduronic acid residues, neighboring α-GlcNS units, are more intense in the decasaccharide derived from HBI than those from HPI and HABH (signals I2-C → I5-C) (Figure 8F,G).

## Discussion

NMR techniques are usually used in structural study of the LMWH.^17,18^ The comparative analysis of enoxaparins derived from different heparin sources revealed significant structural variations. While enoxaparins from HBI and HPI exhibited general similarities in 1D ^1^H NMR spectra, specific differences emerged in distinct regions, particularly emphasized by more detailed analyses using 2D ^1^H–^1^H TOCSY spectra. Enoxaparin from HBI displayed additional spin systems of unsaturated hexuronic acid (Δ4,5UA) at the nonreducing end named Uc, linked to α-GlcNS. This structural divergence was mitigated by an additional purification step of the starting material, resulting in a HABH enoxaparin with a 1D ^1^H NMR spectrum resembling that of the product from HPI.

Subsequent analysis using 2D ^1^H–^13^C HSQC spectra provided insights into anomeric signals, highlighting subtle differences in the constituent units of enoxaparin from the three heparin sources. Notably, the elevated presence of α-Glc*N*S (without 6-sulfation) in enoxaparin from HBI was a marked distinction (HBI > HABH > HPI). Other discrepancies were observed, such as the higher 2-sulfation content of α-IdoA in HBI and HABH compared to HPI. This is consistent with bovine-origin heparins and persists in HABH, as previously demonstrated.^13^ The intensity of the A6 signal compared to C6 emerged as a differentiating factor, with HPI > HABH > HBI, reflecting varying proportions of sulfated and nonsulfated α-Glc*N*H units.

Other differences can be observed in Table 2. However, these cannot be attributed to the peculiarities of the β-elimination reaction conducted in our laboratory. Standard enoxaparin (obtained from USP) and our laboratory preparation show no differences in disaccharide composition (HPI-A vs HPI-B).

Analysis of enoxaparin composition, whether determined by 2D ^1^H–^13^C HSQC (Table 1) or by heparitinase digestion (Table 2), yields similar results. Any minor differences are attributed to the specificity of the two methods, such as the inability to differentiate α-IdoA and β-GlcA units by enzymatic digestion or inaccuracies in estimating the content of *N*-sulfate and *N*-acetyl groups by the spectroscopic method.

Additionally, gel filtration chromatography provided valuable insights into the composition and quality of enoxaparin preparations from different sources.^19^ The dissimilarity observed between products generated by HBI and HPI in the β-elimination process underscores the impact of the heparin source on the characteristics of the resulting enoxaparin. Importantly, the limitation associated with the molecular mass distribution of HBI enoxaparin was effectively addressed by using HABH. The resulting chromatogram demonstrates improved resolution, aligning more closely with the expected characteristics of enoxaparin.

Once the physicochemical characterization of the enoxaparins obtained from the three types of heparins was established, the next step was to determine their anticoagulant actions using standardized coagulation assays, as in Figure 6. To further evaluate the biological impact of enoxaparins, their affinity for antithrombin, a crucial target of heparin, was investigated through titration based on the change in intrinsic serpin fluorescence, well-stabilizing method.^20,21^ The results affirm the similarity between enoxaparins obtained from HABH and HPI, while the product derived from HBI exhibits a diminished affinity for serpin, as evidenced by higher *K*_d_ values for HBI. The evaluation of enoxaparins in an experimental model of venous thrombosis in mice corroborates the observed differences in anticoagulant activity. Enoxaparins from HABH and HPI demonstrate similar effects in inhibiting thrombus formation, whereas the product from HBI is notably less effective.

The analysis of oligosaccharides obtained from HBI, HPI, and HABH revealed distinct structural characteristics. They exhibited identifiable spectral regions that were crucial for quantifying their structure. The integration of H1 and H4 signals from the nonreducing terminal units of unsaturated hexuronic acid showed a decrease with increasing DP, as expected. Interestingly, the relative intensities of the ΔU4c and ΔU4a signals remained constant with DP, indicating consistent structural features across oligosaccharides from different sources.

Subsequent analysis focused on decasaccharides purified from enoxaparins derived from the three heparin sources. Analytical gel filtration chromatography confirmed the purity of the preparations, with each one exhibiting a single peak. However, anticoagulant assays revealed significant differences among decasaccharides from different sources, with the HBI-derived decasaccharide showing markedly reduced anticoagulant activity compared to those from HABH and HPI. Notably, intense signals from 6-desulfated Glc*N*S and 2-sulfated iduronic acid residues were observed in the HBI-derived decasaccharide, contrasting with more discrete signals in decasaccharides from HPI and HABH. These findings underscore structural disparities among enoxaparins sourced from different heparins, highlighting the importance of source selection and purification processes in ensuring product quality and therapeutic efficacy.

## Conclusions

In summary, currently, there are well-accepted criteria ensuring the similarity of pharmaceutical preparations of LMWHs, including the demonstration of equivalence in (1) disaccharide composition, (2) molecular mass distribution, (3) the presence of minor but relevant components, and (4) biological activity. A comparison between enoxaparins obtained from HABH and HPI indicates that these criteria were largely met except for slight differences in disaccharide composition. A crucial yet minor component for the anticoagulant action of enoxaparin is the trisulfated α-glucosamine unit (α-Glc*N*,3,6-triS), crucial in heparin binding to antithrombin. This aspect was investigated by titrating enoxaparin with antithrombin, an indirect but accepted principal for assessing the content of these minority units in heparin that confer a high affinity for the serpin. The question of the bioequivalence of these two types of enoxaparins will require future evaluation, with the decision on whether porcine and bovine enoxaparins are different or similar medicines naturally requiring clinical observations.

Beyond these results with clear practical implications, our work outlines essential procedures for evaluating the production of LMWH from a new source. We combined rigorous structural analysis, using spectroscopic and enzymatic methods, with an assessment of the biological effects of the products based on *in vitro* assays and tests on experimental animals. Furthermore, we analyzed how the β-elimination reaction, used to generate enoxaparin, acted on the heparin chain containing α-Glc*N*H residues with distinct sulfation patterns in a similar way. Therefore, we provide a systematic approach to obtain LMWHs from new sources based on solid principles.

## Materials and Methods

### Glycosaminoglycans

Six representative batches of active pharmaceutical ingredients of HPI (Hepamax-S, from Blau Farmacêutica, Cotia, Brazil) and HBI (Heptar, from Eurofarma, São Paulo, Brazil) were combined and utilized to produce enoxaparin. HABH was derived from HBI, following the previously described method.^13^ The standard enoxaparin used was USP. An additional enoxaparin preparation (Versa) was procured from Eurofarma (São Paulo, Brazil).

### Preparation of Enoxaparins

A solution of heparin (10 mL, 100 mg mL^–1^) was gradually mixed with 17.5 mL of benzethonium chloride (200 mg mL^–1^) at pH 6.7 while continuously stirring at 60 °C. The mixture was then agitated for an additional 60 min, with periodic aliquots withdrawn every 10 min to monitor benzethonium chloride consumption. The resulting precipitate was separated from the supernatant via centrifugation and washed four times with water at 60 °C. Subsequently, the precipitate was lyophilized to obtain benzethonium heparinate, which was then solubilized in DMF (5.1 mL) for 30 min in a 40 °C stabilized bath, with constant stirring and sealing. Following solubilization, benzyl chloride (1.7 mL) was intermittently added to the reaction mixture. After 24 h, the solution was cooled to room temperature, and 6.8 mL of 10% sodium acetate solution in methanol was slowly added. The resulting precipitate was filtered, washed with the same sodium acetate solution in methanol, dried in a desiccator for 24 h, solubilized in water, and subsequently lyophilized. The product obtained in the previous step (benzyl ester of heparin) was then solubilized with 25 mL of Milli Q water per gram of solute at 53 °C. After solubilization, a 1.4 M NaOH solution was added in a 1:15 ratio until reaching a final concentration of 87.5 mM. The reaction proceeded for 90 min at 53 °C, and the solution was neutralized to pH 7.0 with 1 M HCl. Following neutralization, the solution was cooled to 20 °C, and NaCl was added to achieve a final concentration of 10%. Finally, 2 volumes of an 80% ethanol solution were added to precipitate the cleaved material (enoxaparin). The precipitate formed overnight was removed by centrifugation, dried, and weighed.^6^

### Analysis of Enoxaparins by Nuclear Magnetic Resonance (NMR)

NMR spectra of enoxaparin were recorded using a DRX 800 MHz spectrometer (Bruker; Billerica, MA, USA) equipped with a triple-resonance probe. Approximately 20 mg of each enoxaparin or 5 mg of purified oligosaccharides was dissolved in 0.5 mL of 99.9% deuterium oxide (Cambridge Isotope Laboratory; Cambridge, MA, USA), and the spectra were recorded at 35 °C with HOD (deuterium oxide) suppression through presaturation. One-dimensional ^1^H NMR spectra were recorded with 32 scans. Phase-sensitive ^1^H–^1^H MLEV17 TOCSY spectra (4046 × 400 points) were acquired with a spin-lock field of 10 kHz and a mix time of 80 ms. ^1^H–^13^C multiplicity-edited HSQC spectra (1024 × 256 points) were acquired with globally optimized alternating-phase rectangular pulses for decoupling (GARP). The ^1^H and ^13^C chemical shifts were calibrated (0 ppm) based on signals from external standards, trimethylsilyl propionic acid, and methanol (both from Sigma-Aldrich), respectively. The spectra were processed using Top-Spin 4.0 software (Bruker).^17,22^

### Disaccharide Analyses with SAX-HPLC Chromatography

Disaccharides were obtained by incubating 10 mg mL^–1^ enoxaparin with 4.0 Sigma units mL^–1^ each of heparinases I, II, and III (all from *Flavobacterium heparinum*; Sigma/Aldrich) in 20 mM Tris HCl for 24 h at 37 °C. The products of these incubations were diluted with distilled water (1:8), and 20 μL of each sample, along with a mixture of standards of heparin disaccharides (from Iduron, Batch BN5), was applied to a SUPELCOSIL SAX1 column (Sigma-Aldrich) coupled to an HPLC system (Shimadzu). The disaccharides were eluted at 0.2 mL min^–1^ through a gradient of 0 → 1.0 M NaCl in deionized water, containing 0.1 M HCl, and continuously monitored by absorbance at 232 nm. Retention times and peak integrals of the disaccharides were calculated using the HPLC software from Shimadzu LC Solution Rel. 1.25 (Shimadzu).

### Molecular Mass Distribution

Molecular mass was determined through analytical gel-permeation chromatography using a set of TSK gel G4000 SW and G3000 SW columns (Tosoh; Tokyo, Japan) connected to an HPLC system (Shimadzu). The columns were calibrated using Heparin Sodium Molecular Weight Calibrants from the National Institute for Biological Standard and Control (batch EN63QG) and the United States Pharmacopeia standards (calibrant A, batch R06720, and calibrant B, batch R06710).^7^

### Anticoagulant Activity

The anti-FXa and anti-FIIa activities were assessed by measuring the hydrolysis of the respective chromogenic substrate in a ThermoMax Microplate Reader (American Devices, CA, USA). The specific activity (UI.mg^–1^) was calculated using parameters obtained from parallel line assays performed with the international reference standard enoxaparin sodium for bioassays batch F0K265^23^

### Determination of the Dissociation Constant for the Binding of Enoxaparin to Antithrombin

We followed a previously described methodology to study the interaction between enoxaparin and antithrombin based on intrinsic fluorescence changes of the serpin induced upon enoxaparin binding.^24^ The dissociation constant (*K*_d_) for the binding of enoxaparin to serpin was calculated from the enhancement of tryptophan fluorescence emission. These data were analyzed by nonlinear regression binding.

### Venous Antithrombotic Assay

Venous antithrombotic activities of the enoxaparins were assessed by inducing the formation of a thrombus in the mesentery veins of mice by laying filter paper soaked with 8% ferric chloride over the vein for 1 min. Then, we monitored the formation and evolution of thrombus during 60 min using an intravital fluorescence microscope (Zeiss; Oberkochen, Germany), as described. The assays were performed following the guidelines of our institution (Federal University of Rio de Janeiro) for animal care and experimentation.^25^

### Fractionation of Oligosaccharides from the Enoxaparins on the Bio-Gel P-10 Column

Enoxaparins (80 mg of each), dissolved in 0.8 mL of distilled water, were applied to a column (2.0 m x 1.0 cm) of Bio-Gel P-10 (90–180 μm mesh, BioRad, Herculs, CA, USA), eluted with a 10% ethanol:1.0 M NaCl solution (1:1, v/v), at a flow rate of 4 mL h^–1^. Fractions were collected each 15 min and checked for absorbance at 232 nm. The fractions containing the oligosaccharides were pooled, lyophilized, and dissolved in distilled water. Thereafter, the fractions were desalting using a Bio-Gel P-2 (90–180 μm mesh, BioRad, 30 cm × 1 cm).^7^

## Supporting Information Available

1D ^1^H NMR spectra of enoxaparins obtained from HBI (A), HPI (B), and HABH (C); 2D ^1^H–^1^H phase-sensitive TOCSY spectra of enoxaparins derived from HBI (A), HPI (B), and HABH (C); ^1^H–^13^C HSQC spectra in the region between 4.3–6.1/90–110 ^1^H/^13^C ppm of the enoxaparins obtained from HBI (A), HPI (B), and HABH (C); and 1D ^1^H NMR spectra of the oligosaccharides purified from the enoxaparins derived from HBI, HPI, and HABH (PDF)
