# Supplementary material for: A Unique Enoxaparin Derived from Bovine Intestinal Heparin: A Single Purification Step of the Starting Material Assures a Bovine Enoxaparin Like the Standard from Porcine Origin
Source: ACS Omega. 2024 May 10;9(21):23111–20. doi: 10.1021/acsomega.4c02128 (PMC11137703; doi:10.1021/acsomega.4c02128)
Supplement: Supplementary file 1 — ao4c02128_si_001.pdf [file ao4c02128_si_001.pdf]

A Unique Enoxaparin Derived from Bovine Intestinal Heparin: A Single Purification Step of the Starting Material Assures a Bovine Enoxaparin Like the Standard from Porcine Origin

Stephan N.M.C.G. Oliveira [1*#], Francisco F. Bezerra[1*#], Adriana A. Piquet[1], Rodrigo A. Sales[1], Gabrielly C. T. Valle[1], Nina V. Capillé[1], Ana M.F. Tovar[1], Paulo A.S. Mourão[1*]

[1]Laboratório de Tecido Conjuntivo, Hospital Universitário Clementino Fraga Filho and Instituto de Bioquímica Médica Leopoldo de Meis, Universidade Federal do Rio de Janeiro, Rio de Janeiro, 21941-913, Brazil;

[#]These authors contributed equally as first authors.

*Corresponding author:
Paulo A. S. Mourão, Hospital Universitário Clementino Fraga Filho, Rua Professor Rodolpho Paulo Rocco 255, sala 4A01, 21941-913, Rio de Janeiro, Brazil (e-mail: pmourao@hucff.ufrj.br).
Stephan N.M.C.G. Oliveira, Hospital Universitário Clementino Fraga Filho, Rua Professor Rodolpho Paulo Rocco 255, sala 4A01, 21941-913, Rio de Janeiro, Brazil (e-mail: nicollasmarcin@gmail.com).
Francisco F. Bezerra, Hospital Universitário Clementino Fraga Filho, Rua Professor Rodolpho Paulo Rocco 255, sala 4A01, 21941-913, Rio de Janeiro, Brazil (e-mail: felipebezerra_ipu@hotmail.com).

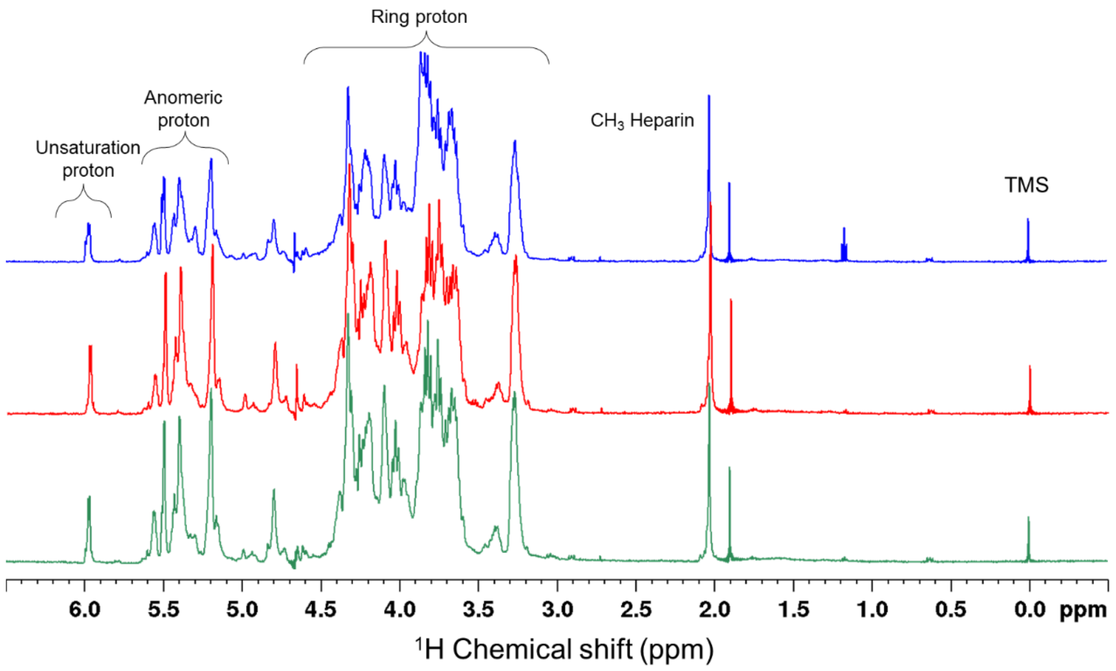

Figure 1S: 1D ^{1}H NMR spectra of enoxaparins obtained from HBI (A), HPI (B), and HABH (C). ΔU1 and ΔU4 are signals from H1 and H4 of the Δ4,5UA units at the non-reducing terminals. A1 and C1 are the anomeric signals of the α-GlcN,6-diS and α-GlcNS units. I1 is the anomeric signal of 2-sulfated α-iduronic acid.

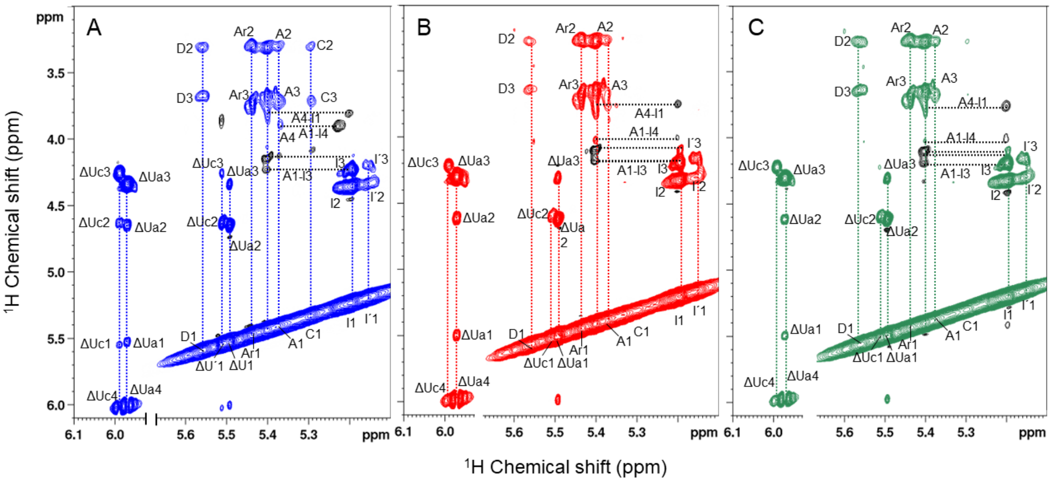

Figure 2S: 2D ^{1}H-^{1}H phase-sensitive TOCSY spectra of enoxaparins derived from HBI (A, in blue), HPI (B, in red), and HABH (C, in green).

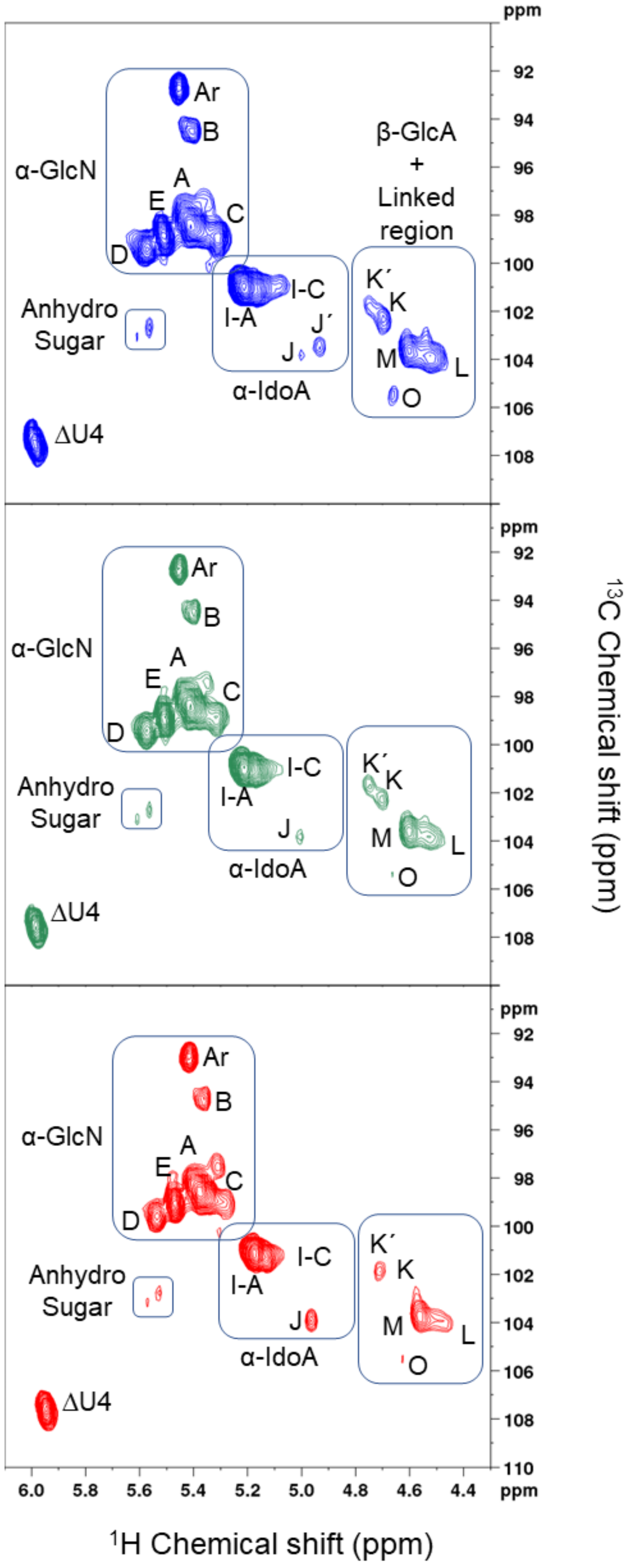

Figure 3S: ^{1}H-^{13}C HSQC spectra in the region between 4.3-6.1/90-110 1H/13C ppm of the enoxaparins obtained from HBI (A), HPI (B), and HABH (C).

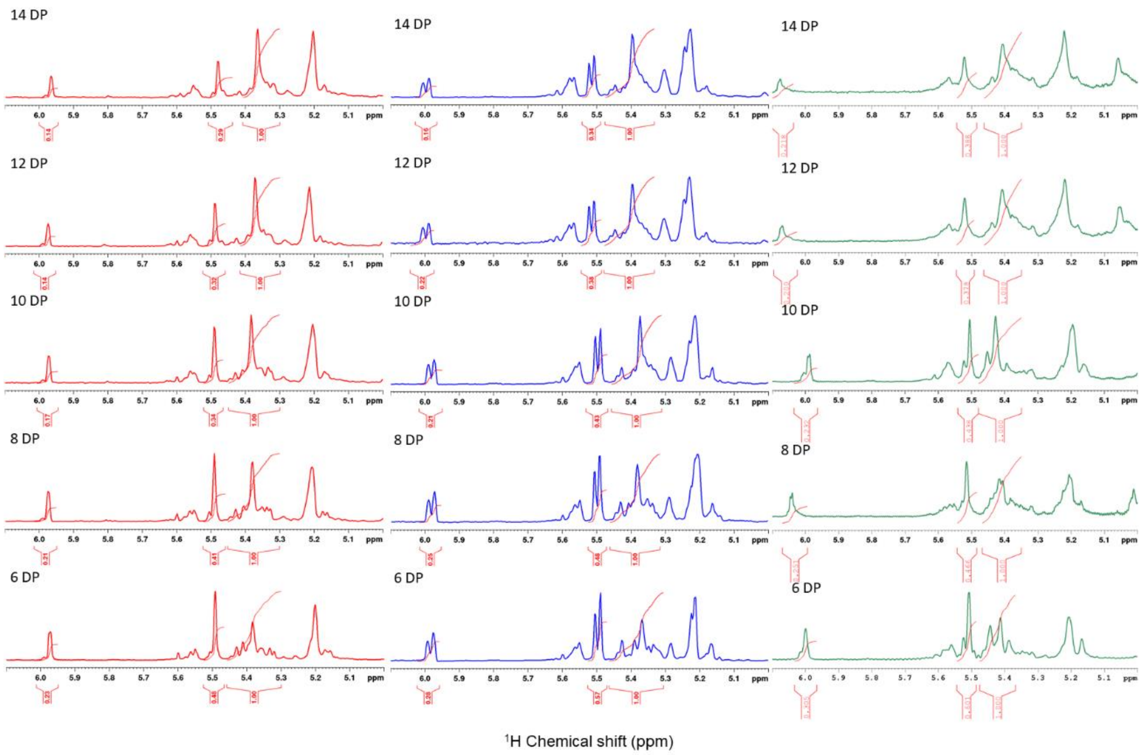

Figure 4S: [1]D 1H NMR spectra of the oligosaccharides purified from the enoxaparins derived from HBI (in blue), HPI (in red), and HABH (in green).
